# *N*-(Phenyl­sulfon­yl)-l-asparagine

**DOI:** 10.1107/S1600536809050247

**Published:** 2009-11-28

**Authors:** Muhammad Nadeem Arshad, Hafiz Mubashar-ur-Rehman, Islam Ullah Khan, Muhammad Shafiq, Kong Mun Lo

**Affiliations:** a Materials Chemistry Laboratory, Department of Chemistry, GC University, Lahore 54000, Pakistan; b Department of Chemistry, University of Malaya, 50603 Kuala Lumpur, Malaysia

## Abstract

In the title compound, C_10_H_12_N_2_O_5_S, one of the sulfonyl O atoms is hydrogen bonded to the amido N atom of an adjacent mol­ecule. There is also a weak hydrogen-bonding inter­action between the other sulfonyl O atom and the secondary amino N atom. In addition, the amido O atom is also hydrogen bonded to a carboxyl O atom. These hydrogen-bonding inter­actions give rise to a layer structure parallel to the *bc* plane.

## Related literature

For related compounds, see: Koroniak *et al.* (2003 ▶); Arshad *et al.* (2008 ▶, 2009 ▶).
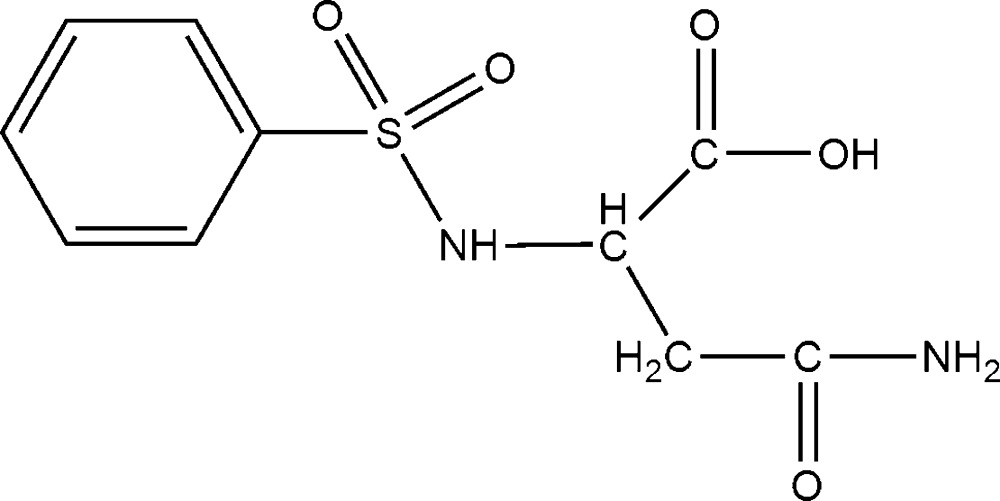


## Experimental

### 

#### Crystal data

C_10_H_12_N_2_O_5_S
*M* *_r_* = 272.28
Monoclinic, 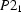

*a* = 10.5479 (6) Å
*b* = 5.1587 (3) Å
*c* = 11.0157 (7) Å
β = 92.011 (3)°
*V* = 599.03 (6) Å^3^
*Z* = 2
Mo *K*α radiation
μ = 0.29 mm^−1^
*T* = 296 K
0.25 × 0.21 × 0.13 mm

#### Data collection

Bruker APEXII diffractometer
Absorption correction: multi-scan (*SADABS*; Sheldrick, 1996 ▶) *T*_min_ = 0.932, *T*_max_ = 0.964
6832 measured reflections
2732 independent reflections
2518 reflections with *I* > 2σ(*I*)
*R*_int_ = 0.023

#### Refinement

*R*[*F* ^2^ > 2σ(*F* ^2^)] = 0.029
*wR*(*F* ^2^) = 0.076
*S* = 1.06
2732 reflections
176 parameters
1 restraint
H atoms treated by a mixture of independent and constrained refinement
Δρ_max_ = 0.33 e Å^−3^
Δρ_min_ = −0.16 e Å^−3^
Absolute structure: Flack (1983 ▶), 1187 Friedel pairs
Flack parameter: −0.01 (6)

### 

Data collection: *APEX2* (Bruker, 2008 ▶); cell refinement: *SAINT* (Bruker, 2008 ▶); data reduction: *SAINT*; program(s) used to solve structure: *SHELXS97* (Sheldrick, 2008 ▶); program(s) used to refine structure: *SHELXL97* (Sheldrick, 2008 ▶); molecular graphics: *X-SEED* (Barbour, 2001 ▶); software used to prepare material for publication: *SHELXL97*.

## Supplementary Material

Crystal structure: contains datablocks I, global. DOI: 10.1107/S1600536809050247/om2295sup1.cif

Structure factors: contains datablocks I. DOI: 10.1107/S1600536809050247/om2295Isup2.hkl

Additional supplementary materials:  crystallographic information; 3D view; checkCIF report

## Figures and Tables

**Table 1:** Hydrogen-bond geometry (Å, °)

*D*—H⋯*A*	*D*—H	H⋯*A*	*D*⋯*A*	*D*—H⋯*A*
N1—H1*B*⋯O1^i^	0.77 (2)	2.31 (2)	3.076 (2)	168.6 (19)
N2—H2*A*⋯O2^ii^	0.95 (3)	2.06 (3)	2.998 (3)	172 (3)
O4—H4*A*⋯O6^iii^	0.82	1.82	2.5804 (18)	155

Symmetry codes: (i) 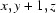; (ii) 
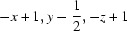
; (iii) 
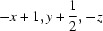
.

**Fig. 1.**
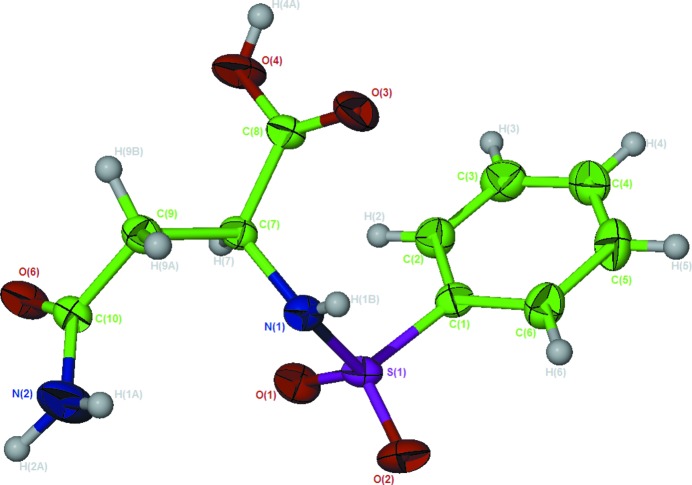
The molecular structure of N-(benzenesulfonyl)-L-asparagine showing 70% probability displacement ellipsoids and the atom numbering scheme. Hydrogen atoms are drawn as spheres of arbitrary radius.

**Fig. 2.**
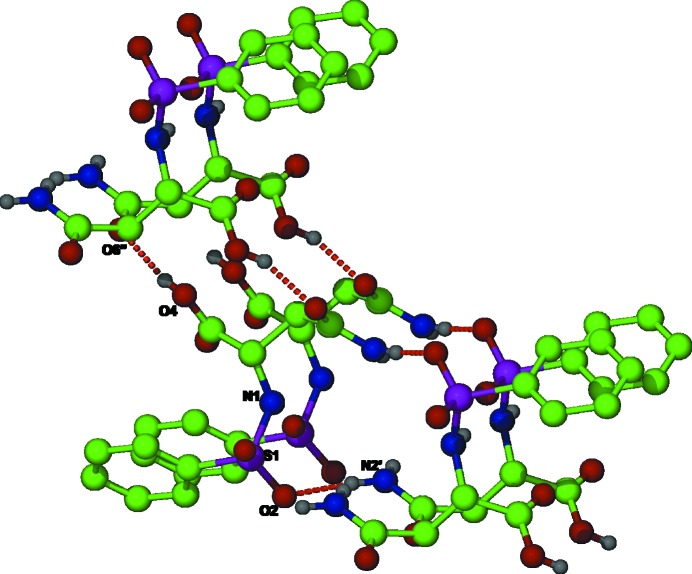
Crystal packing as viewed down the crystallographic b-axis showing the hydrogen bonding interactions. Symmetry code: ii = -x+1,y+1/2,-z.

## supplementary crystallographic information

### Comment

Sulfonamides compounds have long been studied due to their biological activity. *N*-acylsufonamide derivatives of asparagine have synthesized and characterized (Koroniak *et al.*, 2003). The title compound is also a sulfonamide derivative and synthesized in continuation of our studies for the synthesis of acyclic and cyclic sulfonamides (Arshad *et al.*, 2008), (Arshad *et al.*, 2009). In this sulfonamide derivative of L-asparagine (Fig. 1), the molecule is twisted due to the tetrahedral geometries at S1, C7 and C9. One of the sulfonyl oxygen O2 is hydrogen-bonded to the amido nitrogen atom N2 of an adjacent molecule. The amido oxygen O6 also forms hydrogen bond with the carboxylate oxygen O4 of an adjacent molecule. There is a weak hydrogen bonding interaction between the other sulfonyl oxygen O1 and the secondary amino nitrogen N1 (Fig. 2). No significant intramolecular hydrogen bonding interaction is found in the molecule.

### Experimental

Asparagine (0.175 g, 1.32 mmol) was dissolved in distilled water (10 ml) in a round bottom flask (25 ml). The pH of the solution was adjusted at 8–9 using 1*M*, Na_2_CO_3_ solution. Benzenesulfonyl chloride (0.169 ml, 0.234 g, 1.32 mmol) was suspended to the above solution and stirred at room temperature until all the benzenesulfonyl chloride was consumed. The completion of the reaction was achieved when the suspension turned to a clear solution. Upon completion of the reaction, the pH was adjusted 1–2, using 1 N HCl solution. The precipitate obtained was filtered, washed with distilled water, dried and recrystalized in methanol to yield white crystals.

### Refinement

Hydrogen atoms were placed at calculated positions (C–H 0.93 to 0.97 Å) and were treated as riding on their parent carbon atoms, with *U*(H) set to 1.2–1.5 times *U*~eq~(C). The hydroxy H was refined with a restraint of 0.82 (1) Å. The Flack parameter refined to -0.01 (6); there were 1187 measured Friedel pairs.

### Crystal data

**Table:**

C_10_H_12_N_2_O_5_S	*F*(000) = 284
*M**_r_* = 272.28	*D*_x_ = 1.510 Mg m^−^^3^
Monoclinic, *P*2_1_	Mo *K*α radiation, λ = 0.71073 Å
Hall symbol: P 2yb	Cell parameters from 3825 reflections
*a* = 10.5479 (6) Å	θ = 2.6–27.4°
*b* = 5.1587 (3) Å	µ = 0.29 mm^−^^1^
*c* = 11.0157 (7) Å	*T* = 296 K
β = 92.011 (3)°	Block, colorless
*V* = 599.03 (6) Å^3^	0.25 × 0.21 × 0.13 mm
*Z* = 2	

### Data collection

**Table:**

Bruker APEXII diffractometer	2732 independent reflections
Radiation source: fine-focus sealed tube	2518 reflections with *I* > 2σ(*I*)
graphite	*R*_int_ = 0.023
ω scans	θ_max_ = 27.6°, θ_min_ = 2.6°
Absorption correction: multi-scan (*SADABS*; Sheldrick, 1996)	*h* = −13→13
*T*_min_ = 0.932, *T*_max_ = 0.964	*k* = −6→6
6832 measured reflections	*l* = −14→14

### Refinement

**Table:**

Refinement on *F*^2^	Secondary atom site location: difference Fourier map
Least-squares matrix: full	Hydrogen site location: inferred from neighbouring sites
*R*[*F*^2^ > 2σ(*F*^2^)] = 0.029	H atoms treated by a mixture of independent and constrained refinement
*wR*(*F*^2^) = 0.076	*w* = 1/[σ^2^(*F*_o_^2^) + (0.0464*P*)^2^ + 0.0133*P*] where *P* = (*F*_o_^2^ + 2*F*_c_^2^)/3
*S* = 1.06	(Δ/σ)_max_ < 0.001
2732 reflections	Δρ_max_ = 0.33 e Å^−^^3^
176 parameters	Δρ_min_ = −0.16 e Å^−^^3^
1 restraint	Absolute structure: Flack (1983), 1187 Friedel pairs
Primary atom site location: structure-invariant direct methods	Flack parameter: −0.01 (6)

### Special details

**Table:**

Geometry. All e.s.d.'s (except the e.s.d. in the dihedral angle between two l.s. planes) are estimated using the full covariance matrix. The cell e.s.d.'s are taken into account individually in the estimation of e.s.d.'s in distances, angles and torsion angles; correlations between e.s.d.'s in cell parameters are only used when they are defined by crystal symmetry. An approximate (isotropic) treatment of cell e.s.d.'s is used for estimating e.s.d.'s involving l.s. planes.
Refinement. Refinement of *F*^2^ against ALL reflections. The weighted *R*-factor *wR* and goodness of fit *S* are based on *F*^2^, conventional *R*-factors *R* are based on *F*, with *F* set to zero for negative *F*^2^. The threshold expression of *F*^2^ > σ(*F*^2^) is used only for calculating *R*-factors(gt) *etc*. and is not relevant to the choice of reflections for refinement. *R*-factors based on *F*^2^ are statistically about twice as large as those based on *F*, and *R*- factors based on ALL data will be even larger.

### Fractional atomic coordinates and isotropic or equivalent isotropic displacement parameters (Å^2^)

**Table:**

	*x*	*y*	*z*	*U*_iso_*/*U*_eq_	
C1	0.91282 (16)	0.8736 (3)	0.28950 (15)	0.0323 (4)	
C2	0.93349 (19)	0.7209 (4)	0.18909 (17)	0.0411 (4)	
H2	0.8795	0.5838	0.1688	0.049*	
C3	1.03713 (19)	0.7776 (5)	0.11920 (19)	0.0503 (5)	
H3	1.0534	0.6766	0.0516	0.060*	
C4	1.11535 (19)	0.9813 (5)	0.1493 (2)	0.0499 (5)	
H4	1.1837	1.0187	0.1012	0.060*	
C5	1.09413 (19)	1.1314 (5)	0.2501 (2)	0.0517 (5)	
H5	1.1483	1.2683	0.2700	0.062*	
C6	0.99250 (18)	1.0783 (4)	0.32109 (19)	0.0439 (4)	
H6	0.9775	1.1783	0.3893	0.053*	
C7	0.59899 (15)	0.9058 (3)	0.19546 (14)	0.0279 (3)	
H7	0.6111	0.7183	0.1873	0.034*	
C8	0.64948 (16)	1.0412 (3)	0.08415 (14)	0.0311 (3)	
C9	0.45593 (16)	0.9649 (3)	0.20037 (16)	0.0333 (4)	
H9A	0.4444	1.1370	0.2336	0.040*	
H9B	0.4187	0.9630	0.1186	0.040*	
C10	0.38860 (15)	0.7715 (3)	0.27662 (15)	0.0315 (4)	
H1A	0.379 (3)	1.000 (7)	0.406 (3)	0.073 (9)*	
H2A	0.312 (3)	0.722 (7)	0.433 (3)	0.090 (10)*	
N1	0.66143 (14)	0.9939 (3)	0.30846 (13)	0.0300 (3)	
H1B	0.6757 (17)	1.141 (5)	0.3130 (17)	0.027 (5)*	
N2	0.3616 (2)	0.8376 (5)	0.38858 (16)	0.0575 (5)	
O1	0.73791 (14)	0.5586 (3)	0.36312 (13)	0.0448 (3)	
O2	0.79671 (14)	0.9371 (3)	0.49048 (11)	0.0458 (3)	
O3	0.70376 (15)	1.2446 (3)	0.08654 (13)	0.0492 (4)	
O4	0.61999 (16)	0.9065 (3)	−0.01409 (11)	0.0485 (4)	
H4A	0.6403	0.9880	−0.0743	0.073*	
O6	0.35901 (14)	0.5579 (3)	0.23573 (12)	0.0430 (3)	
S1	0.77553 (4)	0.82468 (8)	0.37280 (3)	0.03153 (11)	

### Atomic displacement parameters (Å^2^)

**Table:**

	*U*^11^	*U*^22^	*U*^33^	*U*^12^	*U*^13^	*U*^23^
C1	0.0338 (7)	0.0321 (10)	0.0308 (8)	0.0042 (6)	−0.0010 (6)	0.0009 (6)
C2	0.0425 (9)	0.0410 (10)	0.0399 (10)	0.0005 (8)	0.0027 (8)	−0.0079 (8)
C3	0.0454 (10)	0.0652 (15)	0.0407 (10)	0.0089 (10)	0.0084 (8)	−0.0096 (10)
C4	0.0310 (9)	0.0658 (14)	0.0532 (12)	0.0041 (9)	0.0049 (8)	0.0057 (10)
C5	0.0355 (10)	0.0530 (13)	0.0662 (14)	−0.0071 (9)	−0.0025 (9)	−0.0011 (11)
C6	0.0412 (9)	0.0438 (11)	0.0466 (11)	−0.0015 (8)	−0.0012 (8)	−0.0101 (9)
C7	0.0340 (8)	0.0237 (7)	0.0263 (8)	−0.0001 (6)	0.0039 (6)	0.0008 (6)
C8	0.0357 (8)	0.0312 (9)	0.0268 (8)	−0.0011 (7)	0.0059 (7)	0.0014 (6)
C9	0.0340 (8)	0.0315 (9)	0.0346 (9)	0.0006 (7)	0.0041 (7)	0.0053 (7)
C10	0.0326 (8)	0.0314 (10)	0.0306 (8)	0.0005 (6)	0.0049 (6)	0.0018 (6)
N1	0.0365 (7)	0.0248 (7)	0.0287 (7)	0.0013 (6)	0.0026 (6)	−0.0003 (6)
N2	0.0810 (13)	0.0565 (11)	0.0363 (9)	−0.0212 (12)	0.0216 (8)	−0.0110 (10)
O1	0.0570 (8)	0.0306 (7)	0.0472 (8)	0.0007 (6)	0.0059 (6)	0.0108 (6)
O2	0.0567 (8)	0.0564 (8)	0.0244 (6)	0.0107 (7)	0.0006 (6)	−0.0009 (6)
O3	0.0665 (9)	0.0457 (9)	0.0357 (7)	−0.0255 (7)	0.0061 (6)	0.0041 (6)
O4	0.0820 (10)	0.0388 (7)	0.0255 (6)	−0.0119 (7)	0.0105 (6)	−0.0018 (5)
O6	0.0593 (8)	0.0361 (7)	0.0345 (6)	−0.0074 (6)	0.0152 (6)	−0.0019 (5)
S1	0.0406 (2)	0.0300 (2)	0.02406 (18)	0.00257 (18)	0.00257 (14)	0.00307 (17)

### Geometric parameters (Å, °)

**Table:**

C1—C2	1.382 (3)	C7—H7	0.9800
C1—C6	1.386 (3)	C8—O3	1.195 (2)
C1—S1	1.7596 (18)	C8—O4	1.314 (2)
C2—C3	1.390 (3)	C9—C10	1.499 (2)
C2—H2	0.9300	C9—H9A	0.9700
C3—C4	1.370 (3)	C9—H9B	0.9700
C3—H3	0.9300	C10—O6	1.226 (2)
C4—C5	1.378 (3)	C10—N2	1.320 (2)
C4—H4	0.9300	N1—S1	1.6289 (15)
C5—C6	1.377 (3)	N1—H1B	0.77 (2)
C5—H5	0.9300	N2—H1A	0.88 (4)
C6—H6	0.9300	N2—H2A	0.95 (3)
C7—N1	1.460 (2)	O1—S1	1.4319 (14)
C7—C8	1.523 (2)	O2—S1	1.4304 (14)
C7—C9	1.542 (2)	O4—H4A	0.8200
			
C2—C1—C6	121.60 (18)	O3—C8—C7	124.44 (15)
C2—C1—S1	119.45 (14)	O4—C8—C7	109.93 (14)
C6—C1—S1	118.76 (14)	C10—C9—C7	111.80 (14)
C1—C2—C3	118.21 (19)	C10—C9—H9A	109.3
C1—C2—H2	120.9	C7—C9—H9A	109.3
C3—C2—H2	120.9	C10—C9—H9B	109.3
C4—C3—C2	120.39 (19)	C7—C9—H9B	109.3
C4—C3—H3	119.8	H9A—C9—H9B	107.9
C2—C3—H3	119.8	O6—C10—N2	120.99 (18)
C3—C4—C5	120.86 (19)	O6—C10—C9	120.76 (16)
C3—C4—H4	119.6	N2—C10—C9	118.24 (18)
C5—C4—H4	119.6	C7—N1—S1	120.55 (12)
C6—C5—C4	119.82 (19)	C7—N1—H1B	116.2 (14)
C6—C5—H5	120.1	S1—N1—H1B	110.9 (14)
C4—C5—H5	120.1	C10—N2—H1A	113.9 (19)
C5—C6—C1	119.11 (18)	C10—N2—H2A	117.8 (19)
C5—C6—H6	120.4	H1A—N2—H2A	127 (3)
C1—C6—H6	120.4	C8—O4—H4A	109.5
N1—C7—C8	112.54 (13)	O2—S1—O1	119.35 (9)
N1—C7—C9	108.73 (13)	O2—S1—N1	105.46 (8)
C8—C7—C9	107.92 (13)	O1—S1—N1	106.44 (8)
N1—C7—H7	109.2	O2—S1—C1	108.01 (9)
C8—C7—H7	109.2	O1—S1—C1	109.25 (8)
C9—C7—H7	109.2	N1—S1—C1	107.77 (8)
O3—C8—O4	125.58 (16)		
			
C6—C1—C2—C3	−0.1 (3)	C7—C9—C10—O6	−79.6 (2)
S1—C1—C2—C3	174.80 (16)	C7—C9—C10—N2	100.7 (2)
C1—C2—C3—C4	−0.6 (3)	C8—C7—N1—S1	−99.21 (15)
C2—C3—C4—C5	0.9 (3)	C9—C7—N1—S1	141.28 (13)
C3—C4—C5—C6	−0.6 (3)	C7—N1—S1—O2	−168.94 (12)
C4—C5—C6—C1	−0.1 (3)	C7—N1—S1—O1	−41.22 (15)
C2—C1—C6—C5	0.5 (3)	C7—N1—S1—C1	75.87 (14)
S1—C1—C6—C5	−174.49 (16)	C2—C1—S1—O2	160.50 (14)
N1—C7—C8—O3	−21.2 (2)	C6—C1—S1—O2	−24.43 (17)
C9—C7—C8—O3	98.8 (2)	C2—C1—S1—O1	29.25 (17)
N1—C7—C8—O4	161.31 (14)	C6—C1—S1—O1	−155.68 (14)
C9—C7—C8—O4	−78.71 (17)	C2—C1—S1—N1	−86.01 (16)
N1—C7—C9—C10	−78.10 (18)	C6—C1—S1—N1	89.06 (15)
C8—C7—C9—C10	159.55 (14)		

### Hydrogen-bond geometry (Å, °)

**Table:**

*D*—H···*A*	*D*—H	H···*A*	*D*···*A*	*D*—H···*A*
N1—H1B···O1^i^	0.77 (2)	2.31 (2)	3.076 (2)	168.6 (19)
N2—H2A···O2^ii^	0.95 (3)	2.06 (3)	2.998 (3)	172 (3)
O4—H4A···O6^iii^	0.82	1.82	2.5804 (18)	155

Symmetry codes: (i) *x*, *y*+1, *z*; (ii) −*x*+1, *y*−1/2, −*z*+1; (iii) −*x*+1, *y*+1/2, −*z*.
